# Impact of preparing nursing students to deliver a parent-based sexual health intervention on attitudes and intentions for sexual health education and parent communication counseling: a mixed methods study

**DOI:** 10.1186/s12912-023-01531-2

**Published:** 2023-10-10

**Authors:** Deidra Carroll Coleman, Anitra Frederick, Stanley Cron, Christine Markham, Vincent Guilamo-Ramos, Diane Santa Maria

**Affiliations:** 1 Department of Health Disparities Research, The University of Texas MD Anderson Cancer Center, 1400 Pressler St., Unit. 1440, Houston, TX 77030 USA; 2 Department of Undergraduate Studies, The University of Texas Health Science Center at Houston, Cizik School of Nursing, Houston, TX 77030 USA; 3 Department of Research, The University of Texas Health Science Center at Houston, Cizik School of Nursing, Houston, TX 77030 USA; 4 Department of Health Promotion and Behavioral Sciences, School of Public Health, The University of Texas Health Science Center at Houston, Houston, TX 77030 USA; 5 Center for Latino Adolescent and Family Health, Duke University School of Nursing, Durham, MC 27710 USA

**Keywords:** Adolescents, Family nursing, Nurse education, Parent education, Pediatric nursing, Sexual health

## Abstract

**Background:**

Nurses are well positioned to promote sexual health but are not adequately prepared in their nursing programs to engage families on this topic and often lack the knowledge and confidence necessary to counsel families about sexual health communication. The purpose of this study was to determine how facilitating a parent-based sexual health intervention would impact nursing students’ attitudes and intentions about sexual health education and parent communication counseling.

**Methods:**

Using an embedded mixed-methods design, which integrated a quasi-experimental framework, we examined the impact of participation in a parent-based sexual health intervention among 126 baccalaureate nursing students enrolled in a community/public health nursing clinical course. Independent t-tests, chi-squared tests, and the Mann-Whitney U test were used to compare intervention and control groups at baseline. Multiple linear regression was used to compare the groups for pre-post changes. Qualitative content analysis was used to analyze exit interview transcripts.

**Results:**

We found statistically significant differences in nursing students’ confidence to teach sexual health (*p* = < 0.001), satisfaction with skills as a sexual health educator (*p* = < 0.001), beliefs about the efficacy of parent-adolescent communication for reducing negative sexual outcomes among adolescents (*p* = < 0.001), and intentions to counsel parents on sexual health (*p* = < 0.001), with greater improvements in the intervention group than in the control group. Furthermore, we found statistically significant differences in nursing students’ intentions to counsel parents about the HPV vaccine (*p* = < 0.01) and to endorse the HPV vaccine (*p* = < 0.05), with greater improvements in the intervention group than in the control group. Across all survey categories, qualitative findings confirmed improvements seen on the pre-post survey.

**Conclusion:**

Providing evidence-based adolescent sexual health training, including sexual health education content and discussion strategies, can prepare nursing students to strongly endorse sexual health communication and HPV vaccination uptake and to counsel parents on initiating and navigating these conversations with their youth. Our project exemplifies how a nursing program could organize an immersive experience, or elective within a specialty area, that aligns with the competency-based approach endorsed by the American Association of Colleges of Nursing.

**Trial registration:**

This study was registered with ClinicalTrials.gov (NCT02600884) on 09/01/2015; the first participant was recruited on 09/29/2015.

## Introduction

Adolescents continue to shoulder a disproportionate burden of sexually transmitted infections (STIs) and unplanned pregnancies [1]. To address this disparity, for the past several decades, a plethora of parent-based sexual health education programs have been developed and tested in various communities and settings. Parent-based sexual health education programs are effective at increasing parent–adolescent sexual health (P-ASH) communication, delaying sexual debut, improving condom use, increasing sexual health knowledge and intention to delay sex, and increasing human papillomavirus (HPV) vaccine uptake, especially when delivered to parents of younger adolescents [2, 3]. While some studies have found that a higher intervention dose improved outcomes [3], findings from another meta-analysis suggested that even low-dose interventions were effective [4].

Parent-based sexual health education programs and interventions are delivered by various facilitators, including nurses, pediatricians, social workers, teachers, community advocates, and lay people, as well as online and via computer. While several systematic reviews and meta-analyses of sexual health interventions have been conducted [4–6], none have identified or examined the implications of facilitator type. Additionally, research on the preparation of facilitators and the impact of the training and delivery of these programs on the facilitators is limited. Finally, little information is available on the education and skill building needed to facilitate effective discussions with parents on adolescent HPV vaccination uptake and completion.

Nurses are well positioned to promote the dissemination of P-ASH education in clinics, hospitals, schools, churches, and other community settings [7]. Although nurses and nursing students around the world acknowledge their role in sexual healthcare, they often feel that they are not fully prepared to assume the role of sexual health educator [8]. Across the globe, nursing students have expressed dissatisfaction in their sexual health education preparation and confidence in engaging with families on this topic. For example, Hong Kong nursing students felt that they had inadequate knowledge, were anxious, and were concerned about adverse reactions to sexual health education related to a lack of role modeling of sexual health education [9].

To address this lack of preparation and confidence, nursing education is moving toward competency-based education models with the approval of the new *Essentials* by the American Association of Colleges of Nursing [10]. Therefore, universities and colleges across the United States (U.S) are revising educational models to improve the focus on competency-based evaluation. These changes present an opportunity to better incorporate sexual health education and parent–adolescent counseling and communication strategies into nursing curricula. Nursing students need to be prepared to strongly endorse the importance of, and ideal time for, P-ASH communication [7]. Adequate knowledge, positive attitudes, and communication self-efficacy are positively associated with promoting sexual health care among nursing students [11]. When healthcare providers have access to education to increase their knowledge, practice skills, build self-confidence, and form positive attitudes toward sexual healthcare, they have improved self-efficacy in delivering sexual health education, are more comfortable in delivering sexual healthcare, and more frequently raise the topic with patients [11–13]. Therefore, nursing educators must provide nursing students with the knowledge and skills needed for sexual health education delivery while also helping students form positive attitudes about sexuality to enhance their efficacy in engaging in sexual health education.

Families Talking Together (FTT), an evidence-based intervention, outlines the delivery strategies and content of sexual health topics that should be covered with parents to promote effective P-ASH communication [14–17]. A pilot study in which nursing students completed training on delivering FTT to parents revealed statistically significant improvements in nursing students’ sexual and reproductive health counseling self-efficacy, ability to address barriers to sexual and reproductive health communication, and skills in engaging both adolescents and their parents [18]. In the current study, we examined the impact of nursing students’ participation in an FTT intervention that was adapted to include modules on adolescent immunizations, including the HPV vaccine (herein called FTT + HPV). We sought to determine how facilitating the intervention would impact nursing students’ attitudes and intentions about sexual health education and parent communication counseling. Our secondary aim was to examine the impact of study participation on nursing students’ attitudes and intentions about the HPV vaccine.

## Methods

**Fig. 1:**
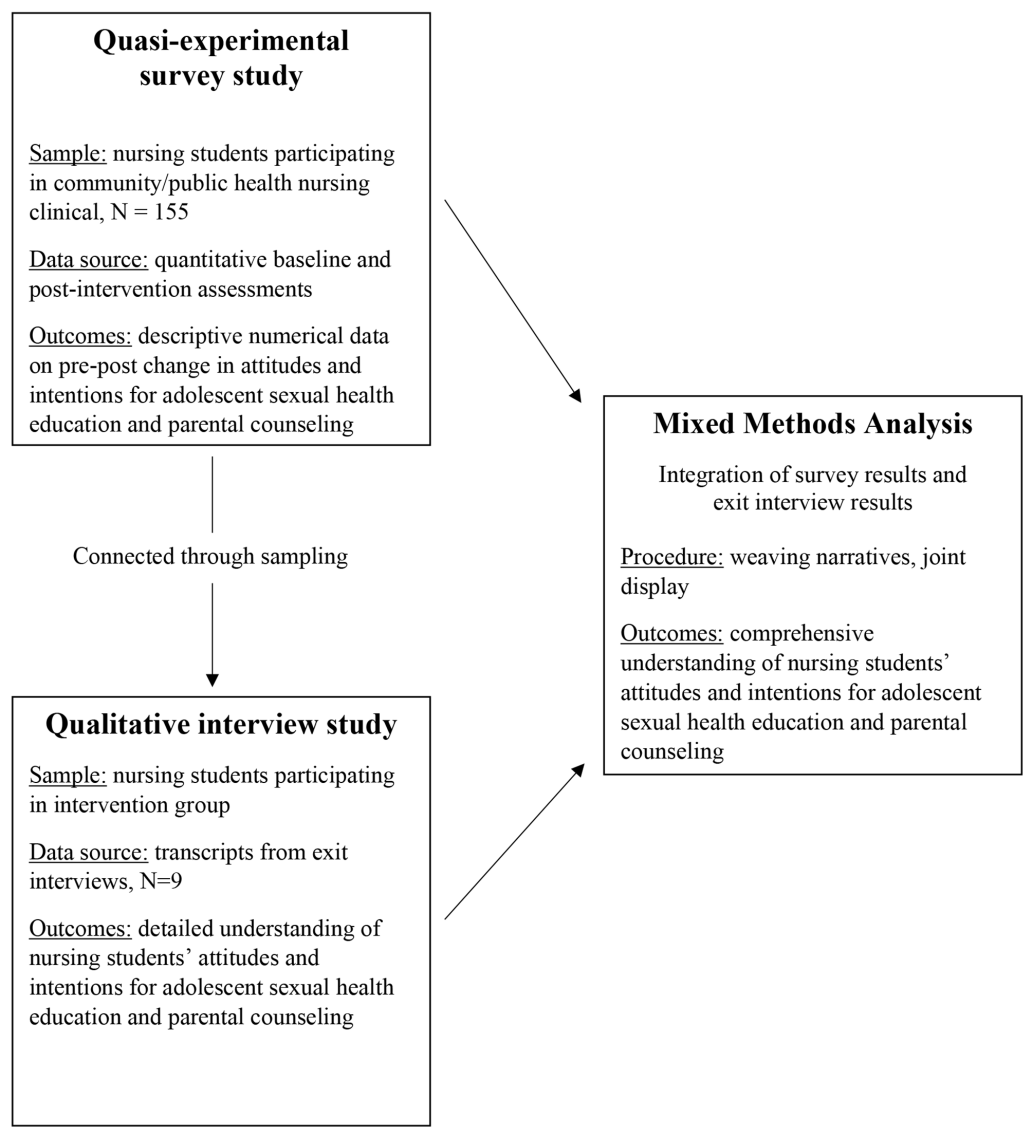
Embedded mixed methods design

**Fig. 2:**
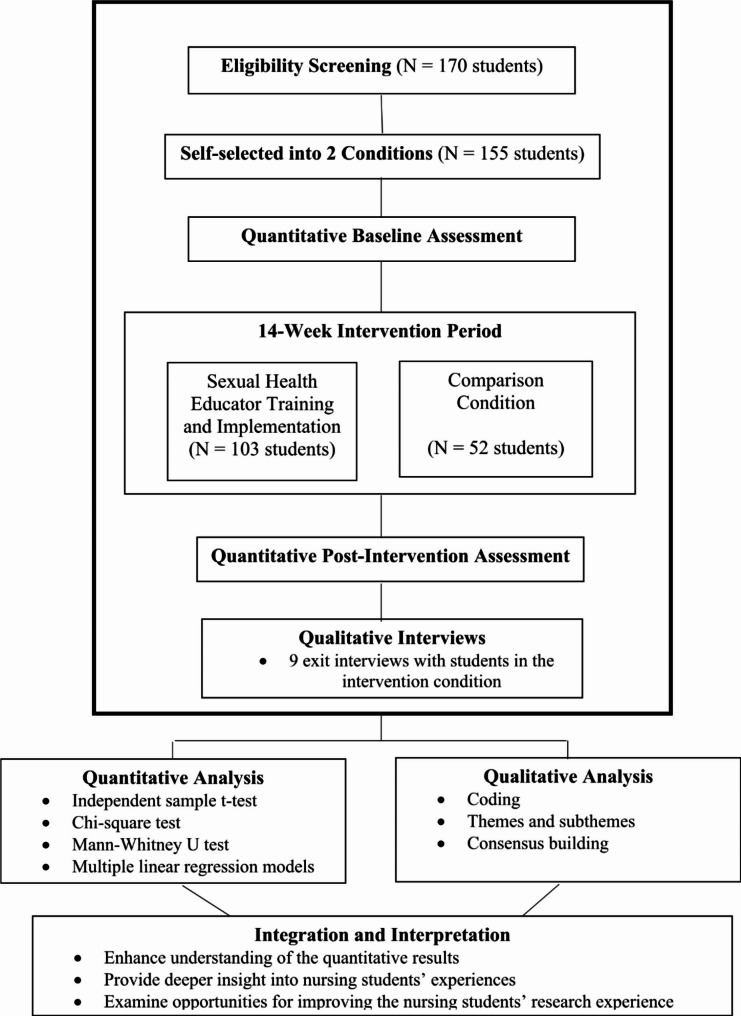
Diagram of mixed methods quasi-experimental study

We employed an embedded mixed-methods design (see Fig. 1), which relied on a primary quantitative dataset to evaluate study outcomes, but used a second qualitative dataset to corroborate study findings [19]. Within this embedded design, a quasi-experimental framework was used to examine the impact of nursing students’ participation in a parent-based sexual health intervention, since randomizing participants was not feasible in this study setting. More specifically, we asked nursing students to complete a pre-test survey. After the 14-week intervention was completed (the typical duration of an academic term, excluding breaks), nursing students in both the intervention and control groups completed a post-test survey; in addition, qualitative methods were integrated to elucidate the survey results of nursing students in the intervention group (see Fig. 2). We integrated quantitative and qualitative methods at three levels: the study design, the study methods, and the interpretation and reporting of the study findings [20].

### Participants

We recruited undergraduate senior-level nursing students enrolled in a community/public health clinical nursing course at a public academic health science center in the southern U.S. between 2015 and 2018 (fall and spring semesters). During this required clinical course, students and faculty work with community partners on promoting health and preventing disease in a target population or area. Students self-select their clinical experience based on their availability or interest in a particular health topic or population.

Students who self-selected an adolescent sexual health clinical were designated to be in the intervention group if they consented to participate in the study. They received 16 h of training on sexual health topics and implementation of the FTT + HPV program. The training consisted of an introduction to adolescent sexual health, STIs, unplanned pregnancy, data on sexual debut and sexual risk behaviors, antecedents of early sexual activity, and parental factors that promote healthy adolescent sexual health development. Students also participated in an in-depth discussion of the principles of effective parent–adolescent communication and provider–parent communication with several hours of practice. Methods used included lecture, group discussion, script development, student presentations, and role playing. After completing the FTT + HPV training, nursing students delivered the intervention to parents in a community-based setting [17].

Students who did not self-select the adolescent sexual health clinical were invited to participate in the study, and if they provided informed consent, they were designated to be in the control group. These students participated in community health clinical experiences that focused on other at-risk populations (e.g., older adults, people with chronic conditions). As part of the usual course requirements, these students collaborated to assess risk, analyze community health problems, plan interventions, and develop a plan for evaluating the interventions.

### Data collection

#### Quantitative data collection

Students in the intervention and control groups completed baseline computer-assisted assessments on tablets at the beginning of the academic semester, prior to attending orientation for their respective clinicals. The baseline assessment included sociodemographic questions as well as measurements of their knowledge, attitudes, and intentions regarding P-ASH education and counseling and HPV vaccination endorsement. Additionally, students completed a matched post-intervention assessment at the end of the semester. Each survey took approximately 30 min to complete.

#### Instrumentation

Confidence in teaching about sexual health topics was assessed with 22 items [21]. Cronbach’s alpha for the Sex Education Confidence Scale was 0.94. Beliefs about P-ASH communication was assessed with five items [22]. Cronbach’s alpha for this measure was 0.65. Perceived adolescent sexual health risk was assessed with five items [14]. Cronbach’s alpha for this measure was 0.70. Barriers to sexual health counseling were assessed with 10 items. Cronbach’s alpha for this measure was 0.79. Satisfaction with skills as a P-ASH educator and counselor was assessed with 10 items [14]. Cronbach’s alpha for this measure was 0.94. Intention to counsel parents on sexual health was assessed with two items developed by the investigators: “I plan to counsel parents about the importance of talking with youth about not having sexual intercourse at this time in his/her life” and “I plan to counsel parents about the importance of talking with youth about protecting him/herself if s/he chooses to have sexual intercourse at this time in his/her life.” Responses were scored on a 5-point agreement scale (strongly disagree, moderately disagree, neither disagree nor agree, moderately agree, strongly agree). Cronbach’s alpha for this measure was 0.94. Beliefs about parental monitoring were assessed with 10 items [14]. Cronbach’s alpha for this measure was 0.86. Intention to counsel parents and adolescents on the HPV vaccine was assessed with two items developed by the investigators: “I plan to counsel parents about the importance of vaccinating youth against HPV” and “I plan to counsel youth about the importance of getting the HPV vaccine at the recommended age.” Responses were scored on a 5-point agreement scale (strongly disagree, moderately disagree, neither agree nor disagree, moderately agree, strongly agree). Intention to endorse the HPV vaccine was assessed with two items developed by the investigators: “How often will you recommend the HPV vaccine to 11- to 12-year-old girls as part of their routine care?” and “How often will you recommend the HPV vaccine to 11- to 12-year-old boys as part of their routine care?” Responses were scored on a 5-point scale (never, rarely, sometimes, often, most of the time).

HPV vaccine endorsement skills were assessed with eight items [23]. Cronbach’s alpha for this measure was 0.68. HPV vaccination status was assessed by inquiring whether nursing students had received the first dose of the HPV vaccine series. If they had, they were asked if they received subsequent doses in the series (i.e., “Did you receive dose 2 of the 3-dose HPV vaccine series?” and “Did you receive dose 3 of the 3-dose HPV vaccine series?”).

#### Qualitative data collection

At the end of each semester, students in the intervention condition participated in group exit interviews facilitated by doctorally prepared nurses and the faculty member teaching the community/public health clinical course. The primary purpose of the exit interviews was to understand nursing students’ experiences with P-ASH education and counseling and to assess their attitudes and intentions for delivering sexual health education in the future. The facilitators used a semi-structured exit interview guide that included questions about students’ overall experiences with adolescent sexual health education, including HPV vaccination knowledge and parental counseling; confidence with adolescent sexual health counseling for parents and youth; beliefs about the importance of adolescent sexual health counseling; and knowledge of effective adolescent sexual health counseling strategies (Table 1). Additionally, the facilitator inquired about students’ perceptions regarding the impact of the clinical experience on their future nursing practice; components of nursing education that prepared students for the clinical experience; which components of the clinical experience should be provided to all nursing students; and aspects of the FTT + HPV training and experience that could be improved.

**Table 1:** Examples of matching survey domains to questions in the qualitative interview guide

Survey questions	Qualitative questions
Confidence in teaching about sexual health topics	Describe your confidence with adolescent sexual health counseling for youth and parents prior to and after this clinical.
Perceived adolescent sexual health risk	Describe how important you thought adolescent sexual health counseling was prior to and after this clinical.
Satisfaction with skills as parent-adolescent sexual health educator and counselor	Describe your knowledge of effective adolescent sexual health counseling strategies prior to and after this clinical

**Table 2:** Nursing Students’ Sociodemographic Characteristics by Group

**Characteristic**	**Total Sample (n = 126)** ^*^	**Intervention** (n = 97)*	**Comparison** **(n = 29)** ^*^	**P Value**
Mean age (SD)	25.5 (5.97)	25.1 (5.53)	26.7 (7.24)	0.23
Ethnicity				0.77
Hispanic	33 (26.2%)	26 (26.8%)	7 (24.1%)	
Race				0.02
White	93 (73.8%)	75 (77.3%)	18 (62.1%)	
Black	13 (10.3%)	6 (6.2%)	7 (24.1%)	
Asian	20 (15.9%)	16 (16.5%)	4 (13.8%)	
Education level				0.98
High school	1 (0.8%)	1 (1.0%)	0 (0.0%)	
Some college	69 (54.8%)	52 (53.6%)	17 (58.6%)	
Bachelor’s degree	46 (36.5%)	38 (39.2%)	8 (27.6%)	
Some grad school	5 (4.0%)	3 (3.1%)	2 (6.9%)	
Master’s degree	5 (4.0%)	3 (3.1%)	2 (6.9%)	

^*^Only includes nursing students who completed both pre-and post-test

### Data analysis

#### Quantitative analysis

Descriptive statistics were calculated for demographic variables and instrument scores at pre- and post-test. Reliability estimates of the instruments were computed with Cronbach’s alpha. Baseline comparisons between the intervention and control groups were conducted with the t-test for independent samples for continuous variables and the chi-square test for categorical variables. The Mann–Whitney U test was used for ordinal variables. Due to a baseline difference in racial composition between the groups, multiple linear regression was used to compare the groups for pre-post changes in instrument scores after adjusting for race. The chi-square test was used to compare the groups for HPV vaccination at each time point. Statistical analyses were conducted with SAS 9.4 for Windows.

### Qualitative analysis

Exit interviews were audio recorded, transcribed by an independent transcription firm, and edited for accuracy. A team member read all exit interview transcripts to gain a general sense of the nursing students’ overall experiences, noted recurring themes, and summarized the preliminary findings. Two team members coded sections of the transcripts that were relevant to adolescent sexual health education, parental counseling, and the HPV vaccine. Team members developed a codebook and coded the remaining transcripts using these codes. We then fit the codes to constructs from the quantitative survey and analyzed quotes to identify recurring subthemes within each construct [24].

### Findings

Table 2 presents sociodemographic characteristics of the nursing students in the quantitative study sample (n = 126). Survey participants were primarily White and had a mean age of 25.5 years old. We conducted a total of nine group-based exit interviews with students from the intervention group. A joint display of quantitative, qualitative, and mixed-methods meta-inferences is presented in Table 3.

**Table 3:** Joint display of quantitative, qualitative, and mixed methods meta-inferences for intervention group

Domain	Change in mean baseline-follow-up	95% CI of diff	Qualitative Findings	Mixed methods meta-inferences
Confidence in teaching about sexual health topics	108–128 ↑20	(15.5; 24.3)	*FTT training session* Before I was very nervous to talk to parents…But then after, it really helped during the training breaking it down in the book and going piece by piece and writing out your script. And at first, I was like maybe that might be a little too—like as if we were reading off of the script, but it really helps when you know the key points that you want to talk about. *Adolescent sexual health education experts* Confidence is really important because even here today, a lot of people said ‘Oh, I felt uncomfortable talking to adults,’ and we all learned that we are adults and we are now these medical professionals that people come to us for advice. *Parent communication counseling experience* Initially, it was really nerve-wracking…majority of us are still on the borderline adolescent age, so we didn’t know how to communicate on like a parent to young adult level. I mean, slowly but surely, as we gained experience, we started to become comfortable in doing that. *Parent engagement* Before clinical, I wasn’t really confident in it. I didn’t think I could do it or would be comfortable doing it. But once we did it actually, it wasn’t really that bad at all. The parents really kind of wanted to know, and that surprised me the most. They were really interested in finding out more about how to talk to their kids. So, I felt a lot more comfortable once we did it.	*Confirmation* FTT training, experience with parental counseling, and perceptions about parents’ engagement in adolescent sexual health education increased students’ confidence, thereby explaining the improvement seen in SECS scores.
Beliefs about parent-adolescent sexual health communication	17–23 ↑6	(4.9; 7.2)	*Parents’ voices matter* Seeing the statistics that parents actually are the voice that teenagers would listen to the most, even if it appears they’re not listening…I have a teenage son, so I’m like, ‘Okay, I’m probably saying things, and it’s going in one ear and out the other,’ but after the training we received, I’m like, ‘Okay, it might appear that way, but something is sticking in there.’ Prior to this, I knew that sexual health was important to discuss…but I felt there were so many barriers… So knowing how to respond to a parent that might say, ‘Well, if I talked to them about it, they’re going to go and have sex,’ telling them that, ‘No, your voice matters…if you teach them your expectations, that actually gives them a guidance of when that time comes, if they’re in that scenario, how they could go about it and make their decision or at least know your opinion about it.’	*Confirmation* FTT training provided evidence for the effectiveness of parent communication counseling for reducing sexual risk-taking in youth, thereby explaining improvement seen in scores for beliefs about parent-adolescent sexual health communication.
Perceived adolescent sexual health risk	23–24 ↑1	(-0.1; 1.4)	*Early sexual debut* Seeing the statistics, that’s a really eye-opening experience because it’s like, ‘wow, these many kids are already having sex.’ *STDs/STIs* I think it helped, feeling burdened by all this information we had to deliver… You want them to reach their goals, and you just want to prevent all these things and STDs. *Teen pregnancy* I knew the topic was important before this experience because a lot of people that I graduated with had their first child when they were like really young but after this and after reading actual statistics and information and stuff, you realize how big of an issue it is.	*Confirmation* FTT training highlighted prevalence of early sexual debut, STD/STI risk among adolescents, and teen pregnancy, thereby explaining increase seen in scores for perceived adolescent sexual health risk.
Satisfaction with skills as parent-adolescent sexual health educator and counselor	25–47 ↑22	(19.7; 24.5)	*Competent* I feel really prepared to have these conversations in whatever practice I go into. Like, I really feel like at least the information and the approach is engrained enough and I’ve done it enough times that I feel like I could effectively communicate that information moving forward.	*Confirmation* Clinical experience adequately prepared students for sexual health education/counseling, thereby explaining the improvement seen in scores for satisfaction with skills as parent-adolescent sexual health educator and counselor.
Intentions to counsel parents on sexual health	9–10 ↑1	(0.8; 1.7)	*Pressing on* It’s not just going through the training – if it’s not something you really want to do it’s going to be tough because…when you [make booster calls] and they hang up on you—if it’s not in you to know that ‘okay, this can happen,’ or ‘I really want to do this,’ or ‘even if this happens I’ll keep going’—you are really not going to do well. *Counseling readiness* It just makes me open minded to all the patients – if they want to bring up a topic or if they nudge towards a certain topic, and they’re kind of embarrassed. I would be open to talking about it now, especially with regards to sexual health and things like that. It just makes me a lot more ready to have that conversation with any patient that is willing or wants to. *Quelling fears* I think it was quite intimidating talking to some parents because I’m not a parent myself. I don’t have kids. What position am I to talk to them about their kid’s sexual health? But giving them those facts and being the informant in this situation and looking beyond that was really important. *Strategic approach* See, I never knew that you could counsel a parent…people always say, ‘You don’t tell parents how to raise a child,’ or that stuff, but after [the	*Confirmation* Students discussed how the FTT training created an urgency to educate and counsel parents, and provided practical strategies for conversing about sexual health, thereby explaining the improvements seen in scores for intention to counsel parents on sexual health.
			clinical] —I realized that sometimes it’s the approach, the way you say it, the way you come about it, they’ll listen—and so with that I was able to see that—okay, there are some conversations that you can have even though it seems as though you shouldn’t have this type of conversation.	
Intentions to endorse HPV vaccine	9–10 ↑1	(0.4; 1.5)	*HPV vaccine advocate* So it’s kind of opened up my eyes, also with health care providers. They’re not pushing for that. And it’s like—I guess we’re going to be advocates for that. If you’re working with a doctor, they’re not pushing for that, then maybe you should have that talk with the parents.	*Confirmation* Learning about lack of vaccine recommendations from HCPs motivated students to be vaccine advocates, thereby explaining the increase in scores intention to endorse HPV vaccine.
HPV vaccine endorsement skills	17–13 ↓4	(-5.6; -2.9)	*Additional training needed* We need more knowledge to talk about it more…we spent so much time on the FTT script, and we all pretty much wrote ‘oh yeah, and HPV’ at the bottom. Since we didn’t go over it as much, you’re questioning yourself, and [the parents] can probably see that so they’re like, ‘do I take her advice or do I not?’ I just think we need more knowledge about it because they do catch you off guard with questions and you’re like, ‘Shoot, I don’t know.”	*Confirmation* Learning about vaccine hesitancy and lacking sufficient knowledge about the vaccine decreased students’ confidence to endorse vaccine, explaining the decrease in scores for HPV vaccine endorsement skills.

### Sexual health education and counseling

Compared to the control group, the intervention group had a greater increase in confidence in teaching about sexual health topics (*p* = < 0.001, Table 4). Interviewed participants described several aspects of the FTT + HPV training and implementation that contributed to their increased confidence: (1) the training bolstered students’ knowledge about sexual health topics and provided strategies for addressing barriers to P-ASH communication; (2) the students had ongoing opportunities to educate parents about adolescent sexual health topics and received feedback on intervention delivery; and (3) the students were recognized by community members as healthcare professionals with expertise in sexual health education. Parents participating in the sexual health intervention were open to, and engaged in, the educational materials provided by the nursing students.

**Table 4:** Sexual health educator training and implementation effects on intervention and comparison groups by domain

Variable	Mean Diff Intervention	Mean Diff Comparison	Estimated Difference (95%, CI)	P value
Confidence in teaching about sexual health topics	19.91	-0.85	20.76 (14.0, 27.51)	< 0.001
Beliefs about parent-adolescent sexual health communication	6.05	0.14	5.92 (4.17, 7.67)	< 0.001
Perceived adolescent sexual health risk	0.68	1.50	-0.82 (-2.02, 0.38)	0.18
Barriers to sexual health counseling	-6.53	-0.60	-5.92 (-8.54, -3.30)	< 0.001
Satisfaction with skills as a parent-adolescent sexual health educator	22.14	2.76	19.38 (15.61, 23.15)	< 0.001
Intentions to counsel parents on sexual health	1.23	-0.31	1.54 (0.80, 2.28)	< 0.001
Beliefs about parental monitoring	2.41	-1.69	4.10 (2.18, 6.01)	< 0.001
Intentions to counsel parents on HPV vaccine	0.38	-0.43	0.81 (0.18, 1.45)	0.01
Intentions to endorse HPV vaccine	0.93	0.02	0.91 (-0.003, 1.82)	0.05
HPV vaccine endorsement skills	-4.25	-0.55	-3.70 (-5.88, -1.52)	0.001
HPV vaccine safety	0.004	-2.03	2.04 (0.77, 3.30)	0.002
Intentions to initiate HPV vaccine	0.51	0.59	-0.09 (-1.04, 0.87)	0.86

Compared to the control group, the intervention group had a greater increase in the belief that P-ASH communication was effective for reducing negative negative sexual health outcomes in youth (*p* = < 0.001, Table 4). Nursing students described being uncertain about the effectiveness of parent–adolescent communication for changing youth behaviors prior to taking part in the community/public health clinical rotation. They credited the intervention with shifting their beliefs and empowering them to counsel parents and counter myths about parent–adolescent communication. Students described a resolve to educate parents about the effectiveness of sexual health communication and hoped that parents who were not ready to have these discussions with their children during the study would someday be willing to have such conversations. Additionally, the intervention group had a greater increase than the control group in their beliefs about parental monitoring (*p* = < 0.001, Table 4). However, this theme did not emerge during the exit interviews.

Perceptions of adolescent sexual health risk did not differ significantly between the intervention and control groups (*p* = 0.18, Table 4). Many nursing students described being aware of risky sexual behavior among youth prior to participating in the study; they recognized the need for adolescent sexual health education programs, which partly motivated the students to select the clinical. For these students, and for the few who had only learned about adolescent sexual health risk during the study, being exposed to statistics about early sexual debut, the prevalence of STIs among young people, and teenage pregnancy rates solidified the magnitude of risk and motivated students to work toward mitigating those risks.

The intervention group had a greater decrease than the control group in barriers to sexual health counseling (*p* = < 0.001, Table 4). Interviewed participants reported perceived barriers to sexual health counseling that were not mentioned in the survey—for example, their age, childlessness, and limited training in adolescent sexual health. In addition, students described several concerns regarding counseling parents, including being viewed as judgmental, discussing taboo topics, and inexperience conversing about sexual health. Interestingly, interviewees described a powerful shift in their perspective of these barriers after recognizing that parents viewed them both as trusted healthcare professionals and young adults with relevant knowledge of the attitudes and behaviors of youth.

Satisfaction with skills as a P-ASH educator and counselor increased more in the intervention group than in the control group (*p* = < 0.001, Table 4). No participants had prior experience or training in sexual counseling and education. However, after the clinical, nursing students described feeling much more competent as sexual health educators. Students discussed the value they place on being nurse educators and, overall, described feeling prepared to engage parents and youth, as well as strangers and family members, regarding adolescent sexual health topics. Nearly all interviewees described having gained a skillset they did not previously have but also acknowledged that their sexual health education and counseling skills could be further improved with additional practice and experience.

The intervention group had a significantly greater increase in intentions to counsel parents on sexual health than the control group (*p* = < 0.001, Table 4). Students described their intentions for providing adolescent sexual health education and counseling in various ways. First, they discussed parental counseling in the context of the clinical experience, emphasizing the importance of educating community members about the benefits of P-ASH communication, even if parents were hesitant to have these conversations with their children. Additionally, interviewees discussed the importance of quelling their own fears and concerns about parental counseling, understanding the repercussions of not educating parents about adolescent sexual health. Finally, students described taking advantage of opportunities to practice their new skills to counsel family members and friends and noted their readiness to counsel parents in informal settings, as well as in their future roles as nurses.

### HPV vaccine

The intervention group had a greater increase in intentions to counsel parents about the HPV vaccine than the control group (*p* = 0.01, Table 4). Students described educating parents about the HPV vaccine, including the recommended age for HPV vaccine administration, the age range for catch-up vaccines, the need to vaccinate both boys and girls, and various HPV-associated cancers that the HPV vaccine might prevent. Additionally, students discussed countering myths about the vaccine when counseling parents.

Additionally, the intervention group had a greater increase in intentions to endorse the HPV vaccine than the control group (*p* = 0.05, Table 4). Students were surprised to learn from parents that some healthcare providers had not recommended the HPV vaccine for their adolescents Students discussed the importance of nurses intervening and making recommending the vaccine when other healthcare providers did not. Students offered strategies for increasing HPV vaccine uptake, including more targeted follow-up for families who have already initiated the vaccine and bundling the HPV vaccine with other recommended adolescent vaccines.

Interestingly, the intervention group had a greater decrease in HPV vaccine endorsement skills than the control group. In general, students thought the HPV component of the P-ASH intervention should be bolstered to adequately prepare students to endorse the HPV vaccine. Students contrasted the emphasis of the training they received for delivering general sexual health content to the training they had received specifically for endorsing the HPV vaccine; additionally, students pointed out that a substantial proportion of the parental counseling session was dedicated to general sexual health content, while only a few minutes were usually spent on HPV education and vaccine endorsement. Despite the training, some students did not feel fully equipped to answer questions or have an informative discussion about the HPV vaccine.

At baseline, 57% of the survey participants reported receiving one dose of the HPV vaccine series, 52% reported receiving two doses, and 42% reported receiving three doses. At post-test, HPV vaccination status did not differ between the intervention and control groups (all p values > 0.61). However, five students in the intervention group reported initiating the HPV vaccine series by the post-test, one student reported receiving dose two, and five students reported receiving the third dose. In the control group, one student reported receiving dose two of the HPV vaccine series by post-test, and one student reported receiving dose three. Additionally, the intention to complete the HPV vaccination did not differ between groups at post-test (*p* = 0.47), although nearly 39% and 22% of the intervention and control group participants, respectively, intended to complete the HPV vaccine series. During the qualitative interviews, we did not inquire whether students had received the HPV vaccine or intended to; however, students discussed their knowledge regarding the HPV vaccine and their experiences with it. In general, the spectrum of student knowledge regarding the HPV vaccine at baseline ranged from “virtually zero knowledge” to having “a lot of education on HPV vaccinations;” however, most students described having a limited familiarity with the vaccine. Some described hearing about it in their obstetrics and gynecology nursing course and from media campaigns, including public health television commercials. Interestingly, no students discussed learning about the vaccine during their own wellness visits. As a result of taking part in the FTT + HPV training and implementation, students recalled learning that both males and females could be vaccinated against HPV, the recommended age range for administering the HPV vaccine (and catch-up vaccines), the HPV vaccine schedule and dosing requirements, and the different types of cancer prevention the HPV vaccine provides. They described the clinical experience as “eye-opening,” and when asked broadly about the impact that participating in the FTT + HPV intervention had in their personal lives, they discussed receiving the HPV vaccine (or intending to) and educating friends and family members about the vaccine.

### Discussion and recommendations

Nursing students who were trained in and delivered an evidence-based sexual health intervention showed significant improvements in their own attitudes, intentions, and skills for adolescent sexual health education and parent communication counseling when compared to students in the control group. Nursing students expressed significantly greater confidence in teaching sexual health topics, noted changes in their beliefs about the benefits of P-ASH communication, and recognized their role as a trusted source of healthcare information. Their intention to counsel parents on sexual health and HPV vaccination was significantly improved as a result of taking part in the intervention. This holds important implications for future nursing practice and the effects of the students’ practice on the communities they will serve. Nursing students can bring about positive changes in communities [17], and while doing so, increase their confidence and intentions to continue using these proven sexual health education interventions in the future.

A secondary aim of our study was to determine the impact of nursing students’ participation in the FTT + HPV training and implementation on their HPV vaccine attitudes, behaviors, and intentions. Nursing students reported an HPV vaccine initiation rate of 57%, which is comparable to the proportion of undergraduate students vaccinated across the U.S. [25]. Additionally, students in the intervention group were not more likely than those in the control group to be vaccinated at post-test. Common barriers among unvaccinated college students are not knowing where to get vaccinated and not having a regular healthcare provider [26]. Campus-based student health service programs using marketing strategies and patient-reminder systems have demonstrated a marked increase in HPV vaccination rates among college students [27] and could be used to increase HPV catch-up for the unvaccinated and under-vaccinated student populations across college campuses.

Nursing education is changing as schools determine best practices for integrating the new *Essentials* [10], a competency-based framework to prepare learners at the generalist level of practice within Four Spheres of Care. Our project, supported by its significant findings of student learning and confidence building, offers an example of how a nursing program could organize an immersive experience or elective within a specialty area. Following instruction from an evidence-based program such as FTT + HPV, competency-based evaluation methods (e.g., simulation scenarios, standardized-patient experiences, and regular demonstration of newly learned skills) could be incorporated, allowing students to obtain a documented area of emphasis upon graduation, such as adolescent sexual health. Similar training procedures and evaluation methods could be applied to virtually any area of interest, allowing students to graduate with specialty knowledge beyond that of the nurse generalist.

Additionally, the *Future of Nursing* report of 2020–2030 calls for strengthening nursing education to improve patient outcomes and healthcare equity [28]. Expanding learning experiences into community settings is highlighted and emphasized by both the American Association of Critical-Care Nurses and the National Academy of Science, Engineering, and Medicine. Aspects of a community-based education intervention shown to provide nursing students the tools and techniques necessary to build knowledge and confidence while practicing effective communication skills could be translated to all areas in which nurses practice.

### Limitations

While our study findings are encouraging, they should be interpreted with caution, as some limitations exist. First, we used a convenience sampling strategy to recruit undergraduate nursing students into the study. Consequently, our sample likely does not represent nursing students across the U.S., and thus, our findings cannot be generalized to the greater nursing student population. Ideally, random sampling, which is the gold standard of sampling strategies, would have been used. However, using a non-probability sample was the most accessible approach for conducting the study and was a logical first step toward determining how facilitating a parent-based sexual health intervention would impact nursing students’ attitudes and intentions about sexual health education and parent communication counseling. Future studies using more robust sampling strategies are warranted given our study findings.

Additionally, students self-selected into groups. Therefore, the intervention group likely comprised students interested in P-ASH communication. Consequently, the group differences seen at post-test could be a result of sampling bias. Even if sampling bias influenced the results, the pre-post improvements in the intervention group should be acknowledged. Ideally, students would be randomly assigned to groups. However, in the current study, this method was not feasible since coursework is sequenced and students are permitted to choose their community health clinical experience based on their personal interests.

Future research should evaluate the impact of nursing students’ participation in sexual health education interventions when students are randomly assigned to various conditions. Furthermore, though all participants were enrolled in a community/public health clinical course, students in the control group likely did not receive intensive training equivalent to that of the students in the intervention group, supporting the evidence that the improvement was associated with the intervention. Finally, social desirability bias may have impacted study findings given that some exit interviews were conducted by the faculty member teaching the community health clinical. Students in the intervention group may have felt the need to please the faculty member as they shared their experiences during group-based exit interviews, which would compromise the validity of the study findings. We did our best to limit the potential for such bias by framing the exit interviews as opportunities to provide feedback for improving the FTT + HPV intervention; however, some students may have still been reluctant to offer constructive criticism. In future studies, exit interviews should be conducted solely by an individual who is not a part of the intervention training and implementation team.

## Conclusions

The future of nursing education is shifting to include a more equitable, community-focused, and competency-based curricula and clinical experience. When provided with evidence-based training, as well as practice opportunities, nursing students are well-equipped to educate and counsel parents in community settings. Furthermore, they have the potential to significantly impact public health in terms of improving the uptake and frequency of protective P-ASH behaviors, which also effect positive changes at the level of nursing students as future healthcare providers.

## Data Availability

The datasets used and/or analyzed during the current study are available from the corresponding author on reasonable request.
